# Service providers’ perspectives on the quality of care of a new complex psycho-oncological care programme in Germany – mixed methods external evaluation results

**DOI:** 10.1186/s12913-026-14854-y

**Published:** 2026-05-30

**Authors:** Natalia Cecon-Stabel, Sandra Salm, Holger Pfaff, Theresia Krieger, Antje Dresen

**Affiliations:** 1 Institute of Medical Sociology, Health Services Research and Rehabilitation Science, Faculty of Human Sciences, Faculty of Medicine and University Hospital Cologne, University of Cologne, Cologne, Germany; 2 Medical Psychology | Neuropsychology & Gender Studies and Centre for Neuropsychological Diagnostics and Intervention (CeNDI), Faculty of Medicine and University Hospital Cologne, University of Cologne, Cologne, Germany; 3 Institute of Medical Sociology, Health Services Research and Rehabilitation Science, Faculty of Medicine and University Hospital Cologne, University of Cologne, Cologne, Germany

**Keywords:** Psycho-oncology, Complex intervention, Evaluation, Quality of care, Mixed methods, Service providers perspective

## Abstract

**Background:**

In Germany, the guideline-compliant provision of psycho-oncological care is challenging. To improve care structures, a newly developed psycho-oncological care programme ‘integrated cross-sectoral Psycho-oncology’ (isPO) was implemented in four psycho-oncological care networks and externally evaluated for quality of care (QoC) and effectiveness. The psycho-oncological service providers’ (SP) perspective was assessed as part of the QoC evaluation.

**Methods:**

A mixed methods design with quantitative and qualitative methods was used. SPs were surveyed twice after programme implementation. They rated their programme satisfaction and contextual and individual factors that might affect its implementation. Medical staff from oncological wards were surveyed once after implementation. Descriptive and correlation analyses were conducted. The independent samples *t*-test was used to compare programme assessment between medical staff and SPs. Semi-structured interviews were conducted with the head psycho-oncologists and two cross-network focus groups with SPs from different professions. One semi-structured interview was conducted for someone who could not attend the focus group. Purposeful sampling was used. All interviews and focus groups were audiotaped, transcribed, and analysed using content-structuring method.

**Results:**

In the surveys, SPs rated their programme acceptance and the care concept positively. Most stated that QoC and cross-sectoral care had improved due to isPO. In the interviews and focus groups, patient benefits were described as the main factors facilitating programme acceptance. Therefore, needs orientation was crucial for structured diagnostics, care intensity, and flexibility in appointment frequencies. Having a fixed contact person across sectorial boundaries was identified as highly beneficial. Organisational workload determinants, such as documentation and bureaucracy, were critically reflected upon as hindering implementation and straining. Participation in quality management activities was perceived as helpful in discussing ways of tailoring the programme to the practice, thereby improving its feasibility.

**Conclusions:**

SPs’ programme assessment and experience help indicate the programme’s feasibility and possible fit for routine care. Including participatory elements in the implementation or development phase may support adapting the programme to an implementation site’s needs. SPs’ experience with the stepped and needs-centred care approach indicates that its underlying concept is recommendable for routine care. However, persistent programme optimisations should be enabled and further implemented within structured quality management.

**Trial registration:**

The study was registered in the German Clinical Trials Register (No. DRKS00015326) on 30 October 2018.

**Supplementary Information:**

The online version contains supplementary material available at 10.1186/s12913-026-14854-y.

## Background

### Psycho-oncological care in Germany

Psycho-oncology deals with ‘the experience and behaviour as well as the social resources of cancer patients in connection with their cancer disease, its treatment and associated problems’ [1]. A guideline-compliant provision and implementation of psycho-oncological (PO) care remains challenging in Germany [2] since only a fraction of cancer patients receive adequate PO care [3, 4]. Utilisation rates may still not meet patients’ care needs, which may be substantiated in multiple aspects: there is a sectoral separation of PO care structures within and between health and social services [5–7], rural areas are underserved [8], there is currently no legal basis for uniform area- and cost-covering financing of PO care [9], and to date, secure funding for the nationwide expansion of cancer counselling centres and qualified counsellors is not ensured [8, 10]. Consequently, the national cancer plan [11] – a coordination and cooperation programme of the German Federal Ministry of Health to further develop and improve the early detection of cancer and the care of cancer patients – calls for integrating PO care into biomedical oncological care and cancer aftercare.

### A new form of PO care - isPO

To meet the national cancer plan’s requests and improve PO care for cancer patients, a new form of care termed ‘integrated cross-sectoral psycho-oncology’ (isPO) was developed, implemented, and evaluated [12, 13] in Germany. This project aims to develop an integrated, cross-sectoral PO programme that is tailorable to different implementation sites. At the patient level, it aims to reduce newly diagnosed cancer patients’ anxiety and depression after one year of PO care.

Care in isPO is provided needs-oriented with a stepped care approach in four newly established PO care networks (CNs) in North Rhine-Westphalia in Germany [12, 13]. A CN comprises a cooperation between at least one certified oncological cancer centre hospital and one oncological practice. First, physicians (e.g. oncologists) refer newly diagnosed cancer patients to the isPO programme. Next, patients are screened for anxiety, depression, and psychosocial risk factors. Based on screening results, patients are allocated to one of four care levels and cared for accordingly (Supplementary Material 1). Various professions are possibly involved in care provision: case managers, trained cancer survivors called ‘isPO onco-guides’, psychosocial professionals, and licensed psychotherapists. The isPO programme also entails quality assurance and improvement structures and a new information technology-supported care documentation and assistance system called CAPSYS^2020^, which supports PO service providers with care documentation, coordination, and billing [13]. Therefore, the isPO programme comprises clinical and formal administrative care aspects [13].

Implementation of the isPO programme’s components started in 2019 in the CNs. In parallel, an independent institute externally evaluated its quality of care (QoC) and effectiveness [12, 13]. Consequently, a comprehensive evaluation study was interlinked with the isPO project [12].

### External evaluation study of the isPO programme

Two frameworks were considered for externally evaluating isPO: The Medical Research Council (MRC)-Framework for the evaluation of complex interventions [14] and the Consolidated Framework for Implementation Research (CFIR) [15]. This approach resulted in a tripartite evaluation design, including a prospective [16] formative and summative evaluation [12]. The summative evaluation assessed the programme’s primary (effectiveness in Hospital Anxiety and Depression Scale [HADS] reduction) and secondary (QoC) outcomes and its transferability [12]. This article will focus on the secondary outcome, QoC; more specifically, focusing on PO service providers’ perspective on the programme. Evaluation results on patient experiences indicate an overall good quality of PO care within the isPO programme [17]. Moreover, the better the QoC was assessed by isPO patients, the better they assessed their health status, work ability, or anxiety and depression levels.

### Objective

We aimed to gain deep insight into service providers’ individual isPO programme experiences and how they assess it. The following overarching research questions arose for the summative evaluation [12]: What are the isPO service providers’ experiences with the programme? What is the isPO service providers’ attitude toward the intervention? What contextual factors promote or inhibit the programme’s implementation?

## Methods

A Mixed Methods Design (MMD) with quantitative (surveys) and qualitative (semi-structured interviews and focus groups) methods was used to assess service providers’ perspectives on isPO’s QoC. Data for the summative evaluation were collected in 2020, the second year of programme implementation.

An isPO-specific Trust Centre was involved in the data collection process since it was responsible to handle sensitive data (including pseudonym lists) under German data protection laws. This procedure was approved by the Ethics Committee of the Medical Faculty at the University of Cologne (No. 18–092).

This article focuses on isPO service providers that are permanent employees and whose roles in isPO entail frequent patient care. Unlike permanent employees, the isPO onco-guides are volunteers and not as integrated into the CNs’ processes and team structures. Therefore, they are excluded from this article. Salm et al. [18] published detailed evaluation results on the isPO onco-guides.

### Surveys

#### Data collection

Within the MMD, service providers were surveyed four months after programme implementation (T1) in their respective CN (in 2019) and then resurveyed 14 months later (T2). The associated Trust Centre sent 300 questionnaires, consent forms, and return envelopes to the respective isPO CN coordinators. The coordinators distributed the documents to the isPO staff at their CN, except for staff working in the cooperating local oncological practices who received questionnaires directly from the Trust Centre. The questionnaires were distributed to all professions possibly involved in the isPO programme or associated with it: medical staff from oncological wards, isPO case managers, isPO onco-guides, isPO psychosocial professionals, and isPO psychotherapists. CN coordinators could also participate. Completed questionnaires and consent forms were directly returned to the Trust Centre. At the request of the evaluation team, the CN coordinators forwarded two reminders for survey participation after two and five weeks. Survey participants provided a contact address (e.g. workplace or home address) for the second survey, ensuring that the network coordinators did not know who was participating. All questionnaires were pseudonymised, which ensured data linkage between the two surveys. The second survey was again organised and processed by the Trust Centre. Participants from the first survey directly received their second questionnaire at their workplace or home address.

From the 300 questionnaires, 56 were completed and returned (response rate: 18.7%). Ten of the 56 participants were isPO onco-guides, who were excluded from this article’s analysis. Of the remaining 46 participants and after two reminders were directly sent out to the service providers by the Trust Centre, 28 returned a completed questionnaire for the second survey (response rate: 60.87%). Data were captured using Teleform and then imported into SPSS (version 29).

#### Data collection – additional survey for medical staff

Only 10 of the 28 participants were medical staff from oncological wards who were not represented in the interviews or focus groups. Therefore, to gather more data on their perspective, a much shorter (2 pages) additional survey was developed and sent out by the Trust Centre in the second year of implementation. Again, 220 questionnaires were sent to the CN coordinators, and 43 completed questionnaires were returned after two reminder rounds (response rate: 19.7%.)

#### Measurements

**Sample characteristics** included education, gender, and employment duration (in years) in the field of activity and the institution. In addition, isPO-specific information is presented, such as the extent of patient care and in what role it is provided.

**isPO programme’s assessment**: Items and scales on isPO service providers’ assessments of the isPO programme itself measured: programme acceptance, programme appropriateness, programme feasibility, satisfaction with the care concept, the enrolment process, HADS utilisation for care level allocation, needs-oriented care due to care levels, feasibility of care paths, satisfaction with isPO-quality circles, usability of CAPSYS^2020^, effort-benefit ratio of isPO and changes through isPO (care quality, patient care beyond hospital boundaries, responsibilities in patient care, early detection of vulnerable cases).

Service providers’ acceptance, appropriateness and feasibility of the isPO programme were measured using the Acceptability of Intervention Measure, Intervention Appropriateness Measure, and Feasibility of Intervention Measure scales [19]. They each comprise four items answered on a five-point Likert scale from ‘do not agree at all’ to ‘totally agree’. Three professional translators independently translated the scales from English into German, translating each item into German and then back into English. A team of four researchers was formed, of which one was a native English speaker with eight years of German language experience. Together, they compared the English translations to the German version. Linguistic subtleties were discussed. For the final German version, linguistic consistency and a comprehensive language were guiding.

Satisfaction with the programme’s care concept was measured with a self-developed single item ‘All in all, what is your personal opinion on the concept of the programme?’. Survey participants could answer on a five-point Likert scale from ‘very bad’ to ‘very good’.

Further, isPO-specific characteristics were measured with self-developed single items that could be answered on a four-point Likert scale from ‘do not agree at all’ to ‘totally agree’. These characteristics included the enrolment process (‘Enrolment/referral of patients to the isPO programme is going well.’), HADS utilisation for care level allocation (‘The classification of patients into care levels with the help of the HADS corresponds to their support needs’), needs-oriented care due to the care levels (‘Through the different care levels of the isPO programme, a needs-based care is possible’), and the feasibility of care paths (‘The predefined care pathways of the isPO programme can be implemented well in the reality of care’). Satisfaction with the isPO-specific quality circles was measured with four self-developed items on their helpfulness (‘The quality circles are proving to be helpful.’), solving upcoming problems (‘The quality circles have helped in solving upcoming problems.’), contributing to optimisations (‘The quality circles contribute to optimisations at our isPO care site.’) and improving communication (‘The quality circles improve communication at our isPO care site.’). Survey participants could answer on a four-point Likert scale from ‘do not agree at all’ to ‘totally agree’.

The System Usability Scale [20] was included in the questionnaire to evaluate the service providers’ perspectives of isPO-specific and newly-developed documentation and assistance system CAPSYS^2020^. Participants could answer the ten items on a 5-point Likert scale from ‘do not agree at all’ to ‘totally agree’.

Survey participants were also asked to provide an overall assessment of the effort-benefit ratio for the isPO programme using a single self-developed item: ‘All in all, how do you assess the programme’s effort-benefit ratio from your point of view?’. Participants could answer on a five-point Likert scale from ‘the efforts significantly outweighed the benefits’ to ‘the benefits significantly outweighed the efforts’.

Lastly, we measured service providers’ assessment of possible changes due to the isPO programme. We asked them to compare their current work situation with the structures and processes before the isPO’s introduction while answering four self-developed items on care quality (‘The quality of care has improved as a result of the isPO programme…’), patient care beyond hospital boundaries (‘Continuous patient care beyond hospital boundaries has improved as a result of the isPO programme…’), responsibilities in patient care (‘Clear responsibilities in patient care have been improved as a result of the isPO programme…’), and early detection of vulnerable cases (‘The early detection of vulnerable cases has improved as a result of the isPO-programme…’). Participants could answer on a five-point Likert scale from ‘very much worsened’ to ‘very much improved’.

**Contextual variables**: In accordance with the MRC Framework for analysing and evaluating complex interventions [14] and the CFIR [15] contextual factors potentially influencing the isPO programme’s implementation were measured. Here, we considered work-related, organisational, and individual variables: Sufficient resources for the isPO, Information needs, Planning deficits [21], Organisational workload, Restrictions through isPO, Changes in work conditions through isPO, Work-related sense of coherence [22], Effort Reward Imbalance Scale [23], Cooperation [21], Trustful organisation [21], Social backing [24], Open communication [21], Organisational readiness to change [25], Resistance to change [26], Personality traits [27] (Supplementary Material 1).

#### Measurements – additional survey of medical staff

In the additional survey of medical staff from oncological wards, the questionnaire included the following items that were also included in the isPO service provider survey: information needs (contextual factor), restrictions at work due to the isPO (contextual factor), and assessment of the care concept (isPO programme assessment).

The following items were only included in the additional survey of medical staff: self-developed single items on knowledge of the isPO programme (‘Had you heard about the isPO programme before this questionnaire?’) and of the programme’s care concept (‘Do you know the programme’s concept?’) with binary answer options (‘yes’ and ‘no’); two self-developed items on patient benefits (‘The isPO-programme benefits patients’) and feasibility (‘The isPO programme can be implemented well’). Participants could answer on a four-point Likert scale from ‘do not agree at all’ to ‘fully agree’.

#### Data analysis

Descriptive evaluations (median, mean, standard deviation, minimum, and maximum) were calculated for all variables. Correlation analyses were conducted with the assessment of the care concept (‘All in all, what is your personal opinion on the concept of the programme?’) and feasibility of the isPO care paths (‘The predefined care pathways of the isPO programme can be implemented well in the reality of care’) as dependent variables. All other variables (isPO-specific and contextual variables) were considered independent variables. Spearman’s rank correlation coefficient (*r*_s_) was used. Due to the small sample, regression analyses were not performed. Data from the separate survey of medical staff was also descriptively analysed. The independent samples *t*-test was used to compare programme assessments between medical staff and isPO service providers.

### Telephone interviews and virtual focus groups

#### Data collection

Qualitative data were collected in the second year of implementation (in 2020). The interviews and focus groups were conducted to gather in-depth insights into the different professions’ opinions and experiences regarding the care programme and its implementation process. We aimed to gain clinical, managing, and coordinating perspectives on the isPO programme. Purposeful sampling [28] was used.

First, the head psycho-oncologists were interviewed and then two focus groups with isPO service providers were conducted cross-network (meaning participants came from different CNs). One person could not attend their focus group and was interviewed afterwards. Due to the coronavirus disease 2019 (COVID-19) pandemic, the interviews (*n* = 4) had to be conducted via telephone and the focus groups (*n* = 2) via a videoconference tool. All qualitative data collections were semi-structured. In addition, the focus groups were guided by a PowerPoint Presentation. Table 1 summarises the data collection and sample details.

**Table 1:** Overview of qualitative data collection methods and the sample

Method	Target group	Number of participants	Role in isPO	Length and tools used
Telephone interview	Head psycho-oncologists	4	Head psycho-oncologists (clinical and managing functions)	Care network (CN) 1: 43 min CN 2: 76 min CN 3: 58 min CN 4: 55 min
Virtual focus group	isPO service providers *(focus group 1)*	7*	Four case managers, of which two deputise for a network coordinator Two psycho-oncologists Two psychosocial professionals, of which one also fulfils the role of an isPO psycho-oncologist	73 min Videoconference PowerPoint presentation
	isPO service providers with managing or coordinating functions *(focus group 2)*	6**	Three network coordinators Three head psycho-oncologists	103 min Videoconference PowerPoint presentation
Virtual interview	Network coordinator who could not attend the focus group 2	1***	Network coordinator	55 Min Videoconference PowerPoint presentation

* Note: only participants from CN 1–3 attended. Participants from CN 4 did not attend

** Note: one care network coordinator who wanted to attend, but had to cancel, agreed to a virtual post-focus group interview

*** Note: the interview was analysed with focus group 2 (managing and coordinating service providers)

The guidelines (Supplementary Material 1) for the interviews and focus groups contain thematic overlaps. The guidelines contain guiding questions on individual evaluations and experiences with the programme’s concept (e.g. ‘How do you experience the exchange at the quality workshops?’ or ‘How do you assess the maturity of the care concept for the respective care levels?’) and questions used to elicit facilitating and inhibiting factors for the programme’s implementation (e.g. ‘How do you experience the implementability of isPO in your care network in regard to facilitating factors?’). In addition, the interviewed service providers were invited to reflect on the extent to which they could imagine a potential transfer to routine national care.

Data collection was conducted by four researchers whose professional background includes qualifications and knowledge from psychology, psychotherapy, nursing, public health, and health services research. All Interviews were audio recorded and transcribed verbatim.

#### Data analysis

Two evaluation team members analysed the data using a content-structuring approach [29, 30] with MAXQDA [31]. Telephone interviews and focus groups were analysed separately with the same procedure: first, a coding system with core categories was developed deductively based on the interview lead questions. Next, the two analysers coded independently, during which inductive categories were derived from the material. The new categories were discussed, and a final category system was agreed upon. Then, a final coding was conducted using the final category system. The content belonging to each category was condensed.

## Results

### Quantitative results

#### Survey of isPO service providers

Of the 28 surveyed isPO service providers (T2), 27 participants reported their role in the project (multiple answers were possible): 18.5% (*n* = 5) worked as isPO case managers, 11.1% (*n* = 3) as psychosocial professionals, 18.5% (*n* = 5) as psychotherapists, 22.2% (*n* = 6) as hospital doctors, 11.1% (*n* = 3) as outpatient doctors, 3.7% (*n* = 1) as a nurse, and 21.4% (*n* = 6) in quality management. Of the survey participants, 28.6% (*n* = 8) worked full-time in the isPO programme, 25.0% (*n* = 7) part-time, and 39.3% (*n* = 11) when needed (e.g. in addition to regular tasks). When the isPO programme started, 21.4% (*n* = 6) of participants had worked in their respective hospital for ≤ 2 years, 17.9% (*n* = 5) for 3–5 years, 21.4% (*n* = 6) for 6–10 years, and 35.7% (*n* = 10) for > 10 years. In addition, 32.1% (*n* = 9) had worked ≤ 2 years in their field, 21.4% (*n* = 6) for 3–6 years, 7.1% (*n* = 2) for 6–10 years, and 35.7% (*n* = 10) for > 10 years. Moreover, 60.7% (*n* = 17) self-identified as female and 35.7% (*n* = 10) as male. The most common educational qualification was the German Abitur (equivalent to a high-school diploma; 60.7%, *n* = 17), and 75% (*n* = 21) had completed a university degree.

Table 2 provides the descriptive results for key variables regarding the programme’s assessment. On average, isPO service providers rated its acceptance, appropriateness, and feasibility from neutral to positive. Comparatively, acceptability and appropriateness were rated better than feasibility. The programme’s concept was rated positively. Assessments of QoC and patient care beyond hospital boundaries have improved due to the isPO programme. The recognition of vulnerable cases and the clarity of responsibilities in patient care have remained similar. The care pathways’ feasibility was rated lowest in comparison to other programme components. The CAPSYS^2020^ system’s suitability was rated as mediocre to good on average.

Most isPO service providers stated that they had no additional information needs for the work tasks, project goals, and care processes. Further, they rated the organisational effort as high and the changes in working conditions through isPO as neutral. Resources (human, personnel, and financial) were rated as insufficient for the programme’s feasibility. Further descriptive results on individual and contextual variables are also provided in Table 2. They were especially considered in the correlation analyses assessing the care concept and care paths’ feasibility (Table 2).

**Table 2:** Descriptive results (counts [n], mean [M], standard deviation [SD]) of variables assessing the isPO programme and its contextual variables and their correlations with satisfaction with the isPO’s care concept and feasibility of care paths

Variable		*n*	M	SD	*r*_s_ satisfaction with the care concept	*r*_s_ feasibility of care paths
**Programme assessment**						
Acceptability of isPO		27	4.13	0.89	0.470*	0.529**
Appropriateness of isPO		27	3.56	0.98	0.503**	0.598**
Feasibility of isPO		26	3.31	0.92	0.662***	0.448*
The isPO care concept		26	3.46	0.99	-	0.531**
System Usability Scale for CAPSYS^2020^		14	69.29	16.77	0.411	0.237
Enrolment process		27	2.48	0.80	0.571**	0.553**
HADS utilisation for care level allocation		26	2.73	0.53	0.321	0.448*
Needs-oriented care due to care levels		27	2.74	0.71	0.460*	0.516**
Feasibility of care paths		26	2.3	0.68	0.531**	-
Satisfaction with the isPO quality circles	– helpfulness	14	2.86	0.77	0.226	−0.500
	– solving upcoming problems	14	2.86	0.70	0.226	−0.360
	– contribute to optimisation in the isPO care network	14	2.93	0.83	0.578*	−0.135
	– improve communication in the isPO care network	14	2.79	0.89	0.228	−0.217
Effort-benefit ratio		27	2.56	1.42	0.645***	0.472*
Changes through isPO	– care quality	27	3.44	1.09	0.729***	0.457*
	– patient care beyond hospital boundaries	26	3.85	0.88	0.521**	0.288
	– responsibilities in patient care	26	3.08	0.89	0.324	0.496*
	– early detection of vulnerable cases	27	3.41	0.84	0.383	0.432*
**Contextual variables**						
Sufficient resources for isPO	– human	27	2.15	0.86	0.072	0.345
	– time	27	2.04	0.71	0.317	0.344
	– financial	27	2.27	0.99	0.460*	0.461*
Information needs	– work tasks	28	2.07	0.86	0.239	0.252
	– care processes	28	2.21	0.88	0.187	0.015
	– project goals	28	1.61	0.79	0.115	−0.225
Planning deficits		28	2.5	0.87	−0.177	−0.374
Organisational workload		28	3.39	0.69	−0.471*	−0.615**
Restrictions through isPO		28	2.57	1.1	−0.634**	−0.596**
Changes in work conditions due to isPO		28	2.89	1.32	0.617	0.564**
Work-related sense of coherence		27	5.02	1.0	0.014	−0.400*
Effort-reward imbalance	– Overcommitment	28	2.29	0.81	0.561**	0.201
	– Effort	28	3.12	0.52	0.160	−0.065
	– Reward	25	2.75	0.63	0.124	0.322
Cooperation between isPO stakeholders		11	3.03	0.46	0.169	0.039
Trustful organisation		27	2.80	0.61	0.435*	0.071
Social backing		26	4.19	0.67	−0.118	−0.188
Open communication		26	2.63	0.66	0.373	0.204
Organisational readiness to change	– change commitment	27	3.37	0.62	0.170	−0.125
	– change efficacy	27	3.11	0.54	0.305	0.032
Innovation climate		25	2.85	0.62	0.411	0.073
Resistance to change	– routine seeking	27	2.31	0.68	0.099	0.402*
	– short-term focus	27	2.24	0.86	0.266	0.383
Personality	– extraversion	27	3.65	0.86	−0.026	−0.234
	– agreeableness	27	3.57	0.58	0.066	0.225
	– conscientiousness	27	3.98	0.70	−0.045	−0.267
	– neuroticism	27	2.7	0.79	0.009	0.136
	– openness	27	5.38	0.77	−0.132	−0.259

*, *p* < 0.05; **, *p* < 0.01; ***, *p* < 0.001

The care concept was more positively assessed the better the assessments of overall programme acceptability, appropriateness, feasibility, the patient enrolment process, needs-oriented care due to the established care levels, care paths’ feasibility, contributing to optimisations at isPO CNs through quality circles, the isPO work-related effort-benefit ratio, changes in care quality, changes in patient care beyond hospital boundaries, financial resources, personal work-related over-commitment, and the organisation’s trustfulness (Table 2). Further, its assessment worsened when organisational workload and work-related restrictions through isPO were high. The feasibility of isPO care paths were more positively assessed the better the assessments of overall programme acceptability, appropriateness, feasibility, care concept, enrolment process, HADS utilisation for care level allocation, needs-oriented care due to care levels, the isPO work-related effort-benefit ratio, changes in care quality, changes in responsibilities in patient care, changes in early detection of vulnerable cases, financial resources, changes of work conditions through isPO, and routine seeking (Table 2). Further, its assessment worsened when organisational workload, work-related restriction through isPO, and work-related sense of coherence were high.

#### Additional survey of medical staff

Of the 43 survey participants, 66.7% (*n* = 28) worked as doctors and 33.3% (*n* = 14) as nurses on oncological wards (one person did not report their profession). In addition, 97.6% (*n* = 41) knew of the isPO programme at the time of the survey.

Most participants reported not having information deficits for programme goals (63.4%, *n* = 26; missings = 2; mean [M] = 2.27, standard deviation [SD] = 0.84) and their tasks/role in the programme (61.9%, *n* = 26; missing = 1; M = 2.33, SD = 0.90). However, half would have liked more information on isPO’s care processes (52.4%, *n* = 22; missing = 1; M = 2.55, SD = 0.77), and more than a third on the programme’s goals (36.6%, *n* = 15) or their tasks/role within it (38.1%, *n* = 16).

In addition, 69% (*n* = 29, missing = 1; M = 2.05, SD = 0.99) of participants reported that their isPO work did not restrict them in daily tasks, while 30.9% (*n* = 13) assessed that it did. Moreover, 92.8% (*n* = 39, missing = 1; M = 3.62, SD = 0.70) believed that the isPO programme was beneficial for patients, and 79% (*n* = 30, missing = 5; M = 3.89, SD = 0.86) rated the programme’s concept as (very) good. In contrast, only 10.5% (*n* = 4) assessed it as bad, and another 10.5% (*n* = 4) as neither good nor bad. Slightly fewer participants (71.4%, *n* = 30, missing = 1; M = 2.83, SD = 0.82) rated the programme’s feasibility as good.

#### Comparison between medical staff and isPO service providers

Compared to isPO service providers, medical staff’s mean information needs for isPO care processes (*t*_(68)_ = 1.68, *p* = 0.049, Cohen’s *d* = 0.409) and programme goals (*t*_(68)_ = 3.47, *p* < 0.001, Cohen’s *d* = 0.847) were significantly higher. Further, medical staff assessed the care concept significantly better than isPO service providers (*t*_(62)_ = 1.86, *p* = 0.034, Cohen’s *d* = 0.473).

### Qualitative results

#### Telephone interviews with head psycho-oncologists

In this section, we consecutively describe head codes that included relevant information on the programme’s QoC: ‘top-down programme development and implementation’, ‘care concept strengths’, ‘care concept weaknesses’, ‘changes because of isPO’, ‘changes because of Corona’, ‘implementability into routine care’ and a subcode under the head code ‘communication in the isPO project’ on the isPO quality workshops. See Supplementary Material 1 for the coding system.

##### isPO quality workshops (subcode: ‘communication in the isPO project’)

The isPO quality workshops played important roles in communicating with the programme’s developers and other CNs. Optimisation processes could be initiated through joint discussions, making isPO more adaptable for practice which was assessed as positive and helpful. However, optimisations took too long to arrive in everyday care, ‘*I find the mills relatively slow*’. The interviewees wished for less ‘*top-down*’ implementation and more ‘*co-determination*’ in the organisation of the quality workshops. The function of the quality workshop as an exchange platform outweighed the function of an information platform.

##### Top-down programme development and implementation

Some interviewees expressed dissatisfaction with the programme development and implementation methodology (too ‘*top-down’ or “scholarly”)* and that programme designers should have considered and used the already existing structures for development: ‘*We have also worked well before and*,* to just sweep it off the desk like that*,* yes*,* of course*,* that doesn’t work like that*.’

##### Care concept strengths

A great strength of isPO’s care concept was seen in its needs-orientation, such as regarding acute or long-term inpatient or outpatient support, the frequency and intensity of the counselling contacts, and the mode of care (i.e. face-to-face or via telephone). It was found to be a great advantage over regular outpatient psychotherapy and enabled patients to receive ‘*no more than necessary*,* but also no less than necessary*’. Demand planning was perceived as ‘*perfect*’, especially for care levels 3a and 3b [note: levels within which patients may see a psychotherapist], and can be organised flexibly. Moreover, early care level allocation facilitated more structured care than before: ‘*Before*,* it was all very wishy-washy*,* the main thing was to talk to them once and then it was somehow OK for the [cancer] centre*’. Structured diagnostics enabled stringent care for patients and the ‘*pre-filtering*’ of individual needs. Overall, isPO’s lower-threshold, needs-oriented care offer was considered meaningful and important in the long term. Therefore, the interviewees suspect that more patients may be reached. Continuity of needs-oriented care across sectors (outpatient/inpatient) was identified as a crucial strength, because patients can have a permanent contact person; they can be supported flexibly for a year, regardless of which setting they are in; and the providers are no longer in conflict regarding billing. This may be beneficial for patients who only develop a need for support after discharge, are in an advanced phase of medical treatment or have short periods of hospitalisation. Therefore, funding outpatient PO care was perceived as the care programme’s greatest ‘*benefit*’ and experienced as a ‘*legitimacy*’ for outpatient services that hardly existed before.

Interdisciplinary patient care was ensured according to the respective staff qualifications, which was rated as very important: ‘*We really need different professional groups that work well together. Then the care levels will really make sense*’.

The care concept was described as comprehensible and ‘*self-explanatory’*. The treatment manual for level 3 care [note: psychotherapeutic care level] may be helpful for newcomers to the profession. The orientation towards a ‘*meta-model*’ [note: the emotion regulation model according to Gross] and the derived intervention suggestions were welcomed. In addition, most of the interviewed head psycho-oncologists evaluated isPO’s onco-guide concept as particularly positive.

Perceptions of the new documentation and assistance system CAPSYS^2020^ were mixed. However, it was positively mentioned that it allows many possibilities to record the counselling sessions’ contents and to get a care overview.

Lastly, the interviewees appreciated that programme optimisations, which were initiated in quality circles and workshops, could be conducted, enabling more practical care: ‘*It was simply much too rigid and strict*,* and it has been gradually adapted*,* which I think is great […] Yes*,* so it is visibly maturing. The cheeks are getting redder and redder.*’. The possibility of being engaged in programme optimisations was experienced as positive.

##### Care concept weaknesses

The care concept was described as theoretical and sometimes lacking orientation to practice, rooted in the programme’s complexity and ‘over-formalisation’: ‘*There is […] a lot of stuff packed in*,* extremely complex*,* they really wanted to include everything that one can understand - of course. […] It is simply too much.*’ As a result, in some cases, implementation was limited in everyday care and felt ‘*imposed*’. Some interviewees expressed that this ‘*over-conceptualisation*’ led to inflexibility, such as in acute patient care, or ‘*artificial products*’ in care path processes, which can slow care. More optimisations were needed to make the programme more practice-oriented, especially at the beginning of its implementation.

It was outlined that the new isPO programme had led to a considerable additional workload for the CNs, sometimes leading to isPO’s tasks being considered infeasible. The added ‘*bureaucracy*’ and the documentation system CAPSYS^2020^ were perceived as particularly burdensome: ‘*[…] everything is added to the normal already busy workday*,* it is extremely difficult to implement for all professional groups. It is simply too much.*’. Some interviewees perceived CAPSYS^2020^ as ‘*over-conceptualised*’ and ‘*research-oriented*’. Its complexity resulted in redundancies, additional documentation and restricted the interdisciplinary communication flow, e.g. with oncologists.

Recruitment was experienced as difficult due to the extent of isPO’s documents, which had to be made comprehensible for patients. Certain patient groups were difficult to reach, for example, patients with low motivation to receive continuous care. Here, telephone appointments proved to be helpful. Moreover, internal hospital factors, e.g. staff shortages supposedly led to lower enrolment, as did the ‘*sluggishness of the system*’, which relates to doctors’ awareness and knowledge of psycho-oncology: ‘*And there are still some who haven’t heard of psycho-oncology […] it has been established for twelve years.*’

All interviewed psycho-oncologists critically reflected on only using screening instruments/questionnaires to allocate patients to a care level. They wished to include the clinical impression in allocating care levels. Moreover, the lack of flexibility in changing care levels was criticised. There were fears of underuse or overuse.

One interviewee gave critical feedback regarding the isPO onco-guide meetings and psychosocial care. The isPO onco-guide meetings were described as difficult to implement and patients’ demand as supposedly low. Patients were experienced as having very specific wishes regarding the characteristics of isPO’s onco-guide (e.g. gender or cancer entity). Since the psychotherapists also fulfil the role of the psychosocial professional in this CN, the interviewee described difficulties separating levels 2 and 3. Regarding content, overlapping appointments were experienced, making the documentation correspondingly difficult.

All interviewees perceived the conceptualisation of care level 3 as very oriented to a behavioural therapy approach, which is also reflected in the documentation system. Other therapeutic approaches were hardly considered, which is reflected in the treatment manual (‘*schematised*’ and ‘*standardised*’). Interviewees expressed that this does not align with everyday therapeutical practice: ‘*So there are certainly some good approaches*,* but there are also some things that need to be seen critically because they are a bit out of touch with the reality of care*’.

Regarding cross-sectoral care, the interviewees stated that the integration of outpatient medical practices has only been implemented to a limited extent and that there is hardly any communication.

Lastly, they wished that patients’ end date for PO care services aligned with their individual needs. When necessary, it should extend beyond one year [note: due to isPO’s study character, care ends after one year of enrolment].

##### Changes because of isPO

From the head psycho-oncologists’ perspective, implementing the isPO programme has changed PO care accessibility (inpatients and outpatients) and needs-orientation in care. Outpatient follow-up care, in particular, has changed positively and ensures continuity of care. However, in some CNs, the inpatient care quality has remained the same or deteriorated in some oncological wards because their existing structures were similar to those of isPO or already functioned well. The increase in the number of patients requiring care in combination with isPO-specific formalisms and a lack of personnel capacity has negatively affected the QoC. However, in some CNs, a greater awareness of psycho-oncology was achieved among medical staff, and the programme component of the isPO onco-guide meetings was evaluated positively.

##### Changes because of corona [COVID-19 pandemic]

At the time of the interviews, the COVID-19 pandemic had already led to changes in care provision within the isPO programme. Interviewees report on a switch from personal to telephone contacts, a decline in enrolment numbers, the cancellation of project meetings (e.g. for cross-network exchange), or the discontinuation of using the isPO onco-guides on the wards since they represented a risk.

##### Implementability into routine care

Regarding the transferability of the isPO programme into standard care, the head psycho-oncologists described some prerequisites and needs for optimisation. Should the programme be implemented nationwide, the care locations structures and personnel resources should be assessed. Regional networks could be used to counteract this problem. Further, it would have to be clarified how outpatient care will be regulated in the long term, such as through a separate ‘*outpatient clinic*’. Another key issue was ‘*convincing*’ the health insurance companies to include the isPO programme in their standard care services.

The importance of an interdisciplinary multi-professional team in PO care was emphasised. At the same time, the number of providers was seen as improvable. Optimisation suggestions also included the addition of the clinical impression for allocating the care levels and less ‘*formalism*’ and ‘*rigidity*’ in the care processes, which in turn are caused by the programme’s current study: ‘*I think it’s great that there’s a project like this*,* but it can also be over-formalised in its approach*,* but then you really have to start rigorously cutting back.*’. The care of relatives and more flexible handling of the start and end of care were also identified as aspects that should be added to the programme.

Finally, psycho-oncology’s relevance for cancer patients’ care was emphasised. Strengthening psycho-oncology by establishing the isPO programme nationwide was seen as important: ‘*Because I believe that […] if we work in a low-threshold way*,* that we work very preventively to a very high degree and […] severe secondary diseases such as […] severe depression or simply our task is also often to improve compliance*,* treatment compliance*,* yes*,* or to maintain it at all by being available for crisis talks*, etc.,* and that this is noticed at all and is also appreciated.*’

#### Cross-CN focus groups

In this section, we summarise the content for the head codes ‘facilitating factors’, ‘hindering factors’, and ‘conditions for adaption into routine care’. See Supplementary Material 1 for the coding system for the focus groups. Focus Group 1 (FG 1) participants included all isPO service provider roles across CNs, while Focus Group 2 (FG 2) participants had managing or coordinating functions within isPO care (Table 1).

##### Facilitating factors

High motivation for programme implementation was expressed. Programme acceptance appeared to have increased, because isPO is better accepted by patients and better understood by service providers (FG 2). In addition, care processes could be implemented well due to the now prevailing routinisation (FG 1): ‘*[…] It takes time to take up care structures*,* and we have now found a way to integrate it really well into the daily routine*’ which positively affected motivation, ‘ *[…] more patients were enrolled*,* it was getting a certain routine. […] you were also motivated by this.*’. Addressing network-specific optimisation requests was perceived as helpful. Cooperation and exchange with the other CNs in the quality workshops was stimulating (FG 2).

Alternative ways of working together were found to improve cooperation with treating doctors, such as the isPO staff taking over certain work steps to relieve doctors. It is believed (FG 1) that the doctors had gained a deeper understanding of PO care through further implementation of isPO.

According to the FG 2 participants, isPO has enabled low-threshold access to psycho-oncology for all patients in their hospitals: ‘*[…] the introduction of the referral forms provides all patients with information that psycho-oncological care is available here at the hospital. We used to have about 600 returns of screening documents*,* and now we have given out 2*,*000 recommendation forms to patients.*’ They assume the optimised patient information materials might have positively influenced patients’ programme acceptance. The service providers (FG 1) perceive it beneficial for patients to receive a cross-sectoral contact person through the isPO programme. The impressions are that patients experience the onco-guide meetings as ‘*very positive’* and therefore have ‘*low-threshold access to self-help groups*’. In one CN (FG 2), the isPO onco-guides were highlighted as particularly beneficial: ‘*The onco-guide worked very well for us […] It is very well thought out here*,* also together with the case managers. It worked really well. […]*’. Also, the offer of psychosocial support is supposedly positively accepted by patients (FG 1): ‘*the support in the psychosocial area is positive in any case […]. It is very*,* very gladly accepted [.] that is actually what I think attracts many patients.*’. The utilisation of CAPSYS^2020^ in routine care was experienced increasingly well after initial optimisation needs: ‘*For the design of CAPSYS*,* however*,* one must rather give the [name of the programmers] a laurel wreath*’.

For the continuation of isPO care during the COVID-19 pandemic, more flexible contact alternatives (telephone instead of face-to-face consultations) were considered helpful (FG 1). The enrolment and information meetings were ‘*mostly easy to implement*’ in this form. FG attendees describe that it was possible to prevent care interruptions, which was essential for patient safety ‘*There was no standstill at all. And that’s really an amazing achievement*,* isn’t it?*’. Moreover, the clarity of roles and work routines in isPO care and the staff’s stability and resilience helped to establish the programme further despite the exceptional circumstances. The isPO programme proved highly relevant for patients during the COVID-19 pandemic since they were more distressed (FG 1) and referrals to psycho-oncology increased: ‘*Some of our patients have also experienced a deterioration of their situation due to Corona. They need us more than ever! So especially those who are not so well integrated socially*,* […] and when the social isolation was added to that*,* many simply felt it in a significantly higher emotional burden*.’. Creating virtual exchange possibilities was considered important for maintaining information flow, patient care, and regular quality circles: ‘*It was good when*,* at least from the isPO side*,* the exchange via these telephone conferences was established […]*’. Organisational factors were also mentioned as influential on implementation during COVID-19 (FG 1). Both options, working from home and from offices, ‘*worked out*’, although the home office arrangement led to the restructuring of work steps. In the beginning, many service providers were ‘*banned from the ward*’ and tried to reach the patients via their room telephones. The isPO onco-guides could also continue working by telephone at this location since they had their own office. At other care sites, case management had more time to concentrate on the enrolment and educational talks because they worked from home offices.

After the first wave of the COVID-19 pandemic in Germany, some service providers gladly utilised telephonic contact options more often (FG 1) as it allowed more flexibility for patients who live far away or want lower-threshold access and some conversations could even be ‘*more open’*: ‘*[…] it’s worked amazingly well […] Sometimes the conversations were even more intense*,* probably because the inhibition threshold is lower*,* so to speak*,* because the other person is not sitting directly opposite.*’. Patient care was now being offered again in a ‘*more holistic*,* structure-guided and isPO-compliant’* way (FG 2) which positively affects work processes and enrolment figures. Despite COVID-19 hygiene rules, a routine has been found in the care process.

##### Hindering factors

A ‘*lack of personnel resources*’ was particularly difficult throughout the isPO project (FG 1). Providing needs-oriented care for increasing numbers of patients was becoming difficult, the bureaucratic effort was perceived as ‘*large and frustrating*’ (FG 2), and the *‘flood of information*’ partially restricted daily work. Reduced staffing and a high workload were associated with stress peaks and, in some cases, even a high level of sick leave (FG 2). Also, the complexity and workload at the hospital management level were underestimated: ‘*we didn’t think about how much extra work that would mean for the other positions. […] we definitely underestimated this effort.*’

It was emphasised (FG 2) that the programme’s adaptation to site-specific needs was insufficient: ‘It is a lot of university culture. That is difficult to implement in a small clinic. You just notice a big discrepancy at this point.’ Further, insufficient CN participation in the isPO-programme’s development phase negatively affected its implementation in the actual care processes. All CNs would have supported an early ‘eye-to-eye discussion’ about the care concept or documentation. The CNs (FG 2) experienced that optimisation requests should have been considered more by programme designers. For instance, some expressed concerns about the screening instruments and emphasised the importance of clinical assessments: ‘[…] it is imperative that our clinical assessment is also recognised as at least as adequate and justifying as the HADS.’. A few service providers (FG 1) were dissatisfied with the assistance and documentation system CAPSYS^2020^. Here, meaning the conceptual incompleteness (e.g., implementation of a module manual) or the ‘ratio of effort and benefit’ was perceived as unsuitable: ‘[…] I still don’t know what I should, should have to, am allowed to do […]’. FG 2 reflected on wishing for problem-centred follow-up training.

All participants emphasised that patients often experienced a bureaucratic overload, especially at the beginning of their cancer treatment, which was intensified by ‘*the study linked to isPO care with all its documents’*. Further, study acceptance and understanding were partly limited oncological staff and isPO service providers, leading to partial rejections. There was often a mixing of care-based and study-based content. A few service providers (FG 1) described that some doctors’ lack of interest in isPO led them not to recommend it to their patients, increasing the work for isPO’s case management (e.g. education and outreach work on the wards). Procedural hurdles and short inpatient stays for patients exacerbated this, potentially hindering quick, needs-based care: ‘*Until the patients […] are able to fill out these questionnaires […]*,* so much time often passes whereas a contact with a psycho-oncologist should have taken place urgently beforehand because it is so necessary […] the process is so […] lengthy.*’

The FG 2 participants reported on the heterogeneity of patients’ care needs, e.g. due to tumour identity, support period, or age. Some patients have a low need, while others ‘*use the service very actively and regularly and basically make and ask for appointments themselves again and again. In some cases*,* they have a significantly higher need than actually envisaged in the project.’* Additionally, the time limit of 12 months was seen as unrealistic and inflexible because many patients were still undergoing treatment: ‘*It can’t be good to conclude at a point in time*,* i.e. to have the final session at a point in time when the* [medical] *therapy is actually not yet finished. Especially in the critical phase*,* I can’t say*,* “Well*,* now the year is over*,* thank you very much and goodbye”.*’

Two head psychotherapists (FG 2) questioned whether the ‘*T3 measurement point for effectiveness analysis* [month 12] *is well chosen*’ since the providers often experience patients’ burden increasing again after month 12 with ongoing therapy. They fear that this may lead to the conclusion that the intervention does not help patients.

The attendees of FG 1 reflected on the different isPO roles and identified optimisation needs: One CN stated that coordinating the isPO onco-guide consultations was challenging and time-consuming, resulting in dissatisfaction. Some found the psychosocial professional (PSF) role conceptually insufficiently distinct from the clinics’ regular social services and wished for a clearly delineated description of the work area. Some psychotherapists found it challenging to distinguish between the roles of psychotherapists providing PO care within isPO and outpatient psychotherapists task areas: ‘*when patients already have mental disorders […] there is an overlap between we don’t do psychotherapy*,* but somehow they are still connected to psychotherapy […] I don’t know exactly what my role is.*’ One psychotherapist also explained that ‘*running after’* patients deviates from their understanding of their profession and role: ‘*[…] I feel like a salesman sometimes […].*’

The COVID-19 pandemic led to diverse changes in the CN. Especially during the lockdowns, the clinics’ care processes shifted: ‘*[…] regular screening was somehow put on hold. […] fewer patients came to us […]. But if they did*,* they were worse off.*’ (FG 2). Some cancer patients appear to suffer particularly from social isolation, while others express clear fears of COVID-19 infection. It was noted that when they came to the doctor, patients were often ‘*much sicker’*. In all, there were fewer inpatient stays since operations were postponed, making fewer patients available for inclusion. While care could be maintained via telephone or virtual care for many patients, others clearly stated that they would rather wait until face-to-face contact was possible again. At first, hardly any recruitment was possible at some CNs (FG 1), ‘*[…] there were simply these enrolment figures in the background*,* […] That’s why […] we’d see to it that we were allowed back on the wards*,* or […] that the outpatients could come to us in the offices again.’.* Some FG participants described the recruitment process as more challenging due to non-visual communication and the complexity of the programme. In some CN, there were delays in the initial care access: ‘*The problem was the feedback from the patients*,* […] everything takes a little longer.*’. A few service providers (FG 1) experienced that their low presence on the wards led to less psycho-oncology awareness among patients. Unfortunately, distributing information via the wards’ management offices could not reduce this effect. Other CNs did not experience such a drastic drop in recruitment. Especially case managers, reported that care demand increased during the COVID-19 pandemic, leading to pressure to process the referral forms quickly, which could hardly be accommodated. In addition, staffing was perceived as too low: ‘*My problem was with staffing levels*,* which were also severely thinned out at the time. Not because of sickness or turnover*,* but shifts […]*’. Some service providers working from home used their private mobile phones. Separating work and leisure time was only possible to a limited extent, leading to a situational burden on the staff. Moreover, limited opportunities for face-to-face exchanges with colleagues led to a feeling of isolation in the working environment, which was perceived as slowing the stabilisation and optimisation of processes in the CN: ‘*[…] it’s a hindrance that sometimes you need the personal exchange in the care or also with all the participants. Yes? […] There’s something missing!*’. In addition, the isPO onco-guide meetings could mostly not be held during the first pandemic wave to protect patients and the isPO onco-guides. For organisational reasons, the switch to telephone conversations was not possible at all CN, so instead case management handed over the information folders to the patients.

##### Conditions for adaption into routine care

Participants from both focus groups identified multi-layered conditions for possibly implementing the isPO programme in routine care. The establishment of comprehensive PO structures was found necessary. Related resources should be built up in all disciplines concerned and a clear concept for implementing the programme after the end of the study should be in place: ‘*I could imagine that there is some kind of additional structure consisting of the relevant professionals. But it is built up independently with spatial*,* material*,* personnel resources*,* in which the patients are connected*,* […] in an outpatient setting.*’ Creating clear transitional regulations to guarantee comprehensive care for cancer patients beyond the project phase was described as important (FG 1). Moreover, adequate financing and providing personnel resources that meet care needs are indispensable for securing a path to routine care (FG 2). Service providers (FG 1) from some CNs reported that hospital management would not promote funding and job creation without increased care services and study success. Therefore, it is particularly important to the providers that psycho-oncology resources are adequately expanded for the programme to be transferred to standard care: ‘*so I think it’s a cycle*,* right? If there are resources*,* you know you can offer them*,* and then they will be accepted*,* and vice versa*,* the thing will certainly become self-perpetuating*.’

The interviewees (FG 2) felt that isPO should be accessible to ‘*all those in need’*, not just newly diagnosed cancer patients. In addition, streamlining information should help guide patients in a more tailored way and promote programme understanding. It was highlighted that flexible, needs-oriented care is particularly important, such as in the care period, care type, and cross-sectoral support services (FG 1). Flexibility regarding stage allocation and start/end of care should also be increased after the end of the study (FG 2) and patient enrolment should be ‘*not only screening-guided’* but combined with clinical anamnesis to optimise needs-oriented care.

The service providers would like to see a reduction in the documentation effort and optimise the documentation instruments’ concept closer to care realities. At the administrative level, the manageability of invoicing appears to be central, ‘*The invoicing is not difficult now*,* but there should be an automatism at the end […] in accounting.’.* Further, the required qualifications of the respective isPO roles should be more clearly defined, especially regarding the role of the psychosocial professional.

From the FG 2 perspective, QoC should be ensured through continuous training, education, and quality circles. Continuous process optimisation should be further conducted, especially regarding the care concept and instruments. It was also emphasised that intersectoral care must be further promoted.

Many focus group attendees addressed the isPO information strategy and overall awareness of psycho-oncology. It was expressed that isPO should be known everywhere it may be offered, and not only by the isPO provider team. They wished for higher acceptance in oncological staff and described that PO services should be treated ‘*like a standard consultation*,* which is registered with every patient who comes to us with an oncological disease*’. Enrolment process could also be simplified or transferred from medical staff to other professions: ‘*We already do it through case management*,* […] so that we can offer it immediately without having to go through the doctor first.*’ In addition, guidelines should strengthen the awareness and relevance of PO care among political lobbying. Awareness of implementing standardised psychosocial support services is perceived as insufficient. At the societal level, it was emphasised that ‘*psycho-oncology must be understood as an integral part of standard oncological care.’* It was expressed that this requires a comprehensive change in health policy culture, not only in the form of ‘*project lighthouses*’: ‘*In terms of health policy*,* there has to be a cultural change! So you still often have the issue that when ‘psycho’ is at the forefront*,* it gets a negative connotation […]*,* it simply has a different effect in society.*’. Finally, it was noted that psycho-oncology as a whole should achieve broader social acceptance among all patients to be effective: ‘*I am even more pleased that in isPO*,* we actually have some men who participate. Otherwise*,* there was a greater rejection of our psycho-oncological care: “I can handle it”*,* or “If I don’t talk about it*,* the problem won’t exist.’.*

## Discussion

In summary, the implementation of the isPO programme is characterized by a high level of acceptance and perceived patient benefit, while simultaneously facing significant structural and bureaucratic challenges regarding its feasibility. To provide a clear overview of these multifaceted findings, Table 3 synthesizes the key quantitative results and qualitative insights across the core dimensions of programme acceptance and feasibility, with further methodological details on scales and codes provided in the Supplementary Material 1.

**Table 3:** Synthesis of key findings on programme acceptance/satisfaction and feasibility of the isPO programme

Topic	Key quantitative findings	Key qualitative findings
Programme acceptance/ satisfaction	Majority of medical staff believe isPO is beneficial for patients. Acceptance was rated higher than feasibility. Satisfaction with the care concept correlates strongly with perceived improvements in care quality.	High appreciation for needs-oriented care *(“no more than necessary*,* no less than necessary”*). Cross-sectoral continuity is seen as a major “legitimacy” for outpatient services.
Feasibility	Resources (human, personnel, financial) were rated as insufficient for the programme’s feasibility. High organizational workload correlates with lower feasibility of care paths.	Perceived “over-conceptualization” and high bureaucratic burden (e.g., CAPSYS^2020^) hinder daily routines. Providers wish for more “co-determination” and the inclusion of clinical impressions in care level allocation.

### QoC in isPO

By applying a MMD, we were able to evaluate how isPO service providers assess the programme’s QoC and gain insight into their personal experiences with implementing the programme. In the survey, isPO service providers rated their programme acceptance and the care concept positively. In the interviews and focus groups, it became clear that patient benefits were a primary factor facilitating programme acceptance because they improved QoC. The term ‘needs-orientation’ was used especially often and related to different topics: structured diagnostics allowing for patient-centred care level allocation, care intensity according to individual distress level by an interdisciplinary team, flexibility in inpatient and outpatient care settings and appointment frequencies, appointment lengths, or modes of care delivery (telephone, virtual, or face to face). Further, having a fixed contact person across sectorial boundaries was identified as highly beneficial for patients. These factors align with the German S3 guideline for PO care and meet the requests of the national cancer plan to facilitate integrated and need-oriented care [1, 32].

Service providers added that the care timeframe should also be managed flexibly, which was not possible due to the study phase of the programme. Some noted that patients tend to have further care needs after they finish their biomedical treatment, which may occur after the one-year period. Studies suggest that while anxiety and depression levels often decrease over time, they are influenced by treatment-related factors (e.g. treatment type, side effects, and prognosis) or patient-related factors (e.g. age, social support, and gender) [33–35]. Therefore, as the isPO service providers suggested, aligning the care timeframe with each patient’s needs appears important.

Survey participants mostly assessed that care quality had improved compared to pre-existing PO structures due to isPO. In addition, care beyond clinical boundaries (cross-sectoral care) has improved. This view is congruent with what interviewees and focus group participants reported. Further, they experienced that awareness of PO care had risen in oncological wards and, as a result, in patients. This result aligns with research findings suggesting a reciprocal relationship between physicians’ and patients’ subjective norms and information deficits and the integration of PO care into routine oncology [36]. While individual norms may act as a barrier to implementing integrated PO care, fully integrating such structures into oncological care increases physicians’ and patients’ acceptance and reduces stigmatised perceptions of PO care [36, 37]. Compared to inpatient oncological wards, isPO service providers reported having little contact with local outpatient oncological practices from the CNs. They wished for a stronger inclusion and engagement of those practices in the network. The stronger inclusion of outpatient oncological practices appears recommendable to avoid fragmentation and further improve the integration of PO care structures.

Service providers’ individual factors, such as the tendency to overcommit, high motivation, identification with programme goals, and social aspects regarding trustfulness in the organisation (trust, working climate, and togetherness feeling), positively influenced the programme’s perception and acceptance. Those individual factors might give the service providers work-related meaningfulness, consistent with the results from the formative evaluation phase [38]. In addition, they may buffer work-related manageability [38], which might have been important because the isPO programme’s feasibility was perceived as mixed. The service providers critically reflected upon the organisational workload for documentation, bureaucracy, and specific care paths. They found those aspects hindered implementation and straining because they increased workload while financial resources were perceived as insufficient. Some of those aspects were substantiated by the programme being in a study phase. In addition, service providers experienced that more patients accessed PO care. However, personnel resources were not always sufficiently available to address these demands, which partially led to distress in service providers. They found it helpful to participate in quality management actions, especially the cross-networks quality workshops, to discuss ways of tailoring the programme to the practice, thereby improving its feasibility. Being able to initiate optimisation processes and communicate with other CNs was perceived as empowering and useful. Therefore, stakeholders’ participation and engagement might be considered helpful [39] in improving a complex intervention programme’s acceptability, feasibility, and effectiveness [40]. This conclusion corresponds to research suggesting that social networks and communications play an important role in implementation processes [15, 41]. Further, Luig et al. [42] stated that the comprehensibility of intervention happens at the collective and individual levels, where information is integrated with pre-existing approaches, the clinical context, and individual experiences. This process is initiated and maintained by conducting the isPO quality circles and workshops and may improve interdisciplinary cooperation, team relationships, shared decision-making, and self-efficacy [42].

Service providers emphasised that while they found structured diagnostics of patients important for care level allocation, the individual clinical assessment of the service providers (e.g. psycho-oncologist) must also be included. When completing a questionnaire, social desirability or other biases may affect patients’ distress assessment [43]. Therefore, it is recommendable not solely to use screening instruments for care level allocation but allow for clinical assessments of professional service providers to ensure needs-oriented care.

For future quality management, service providers want clearer definitions and specifications on qualifications and task areas of the different isPO roles, such as for psychosocial professionals. In some CNs, individuals held two roles (i.e. psychotherapist and psychosocial professional), which may have led to low separability and lower work-related comprehensibility. Work-related comprehensibility is the extent to which work is perceived as clear, structured, and consistent [44]. A low level of it is associated with higher job demands and fewer job resources [22]. Therefore, ensuring work-related comprehensibility may facilitate the implementation process of a complex intervention, such as isPO [38].

Further, psychotherapists wished for the integration of different therapeutic approaches, not just behavioural therapy, which predominantly prevailed in the care concept, manual, and documentation for high care levels. This request aligns with current evidence and as-is status in practice [1, 45].

The experiences with implementing the isPO onco-guides at the CNs were also mixed. Some CNs highly appreciated this new role for patient care and saw it as an improvement for QoC and an opportunity for low-threshold access to self-help services. Studies on one-to-one peer support suggest positive effects on patient health outcomes and self-efficacy [46–48]. Those patient benefits appear also to be visible to the isPO service providers. However, some experienced the underlying organisational workload as too complex or high, negatively affecting the overall perceived benefit. Again, this indicates that practical feasibility and contextual factors may play important roles in implementing the programme. More detailed evaluation results for the isPO onco-guides are published separately [18].

Overall, service providers‘ assessment of the isPO programme’s QoC was split regarding aspects positively affecting patient care (e.g. care continuity, cross-sectoral care, or low-threshold access to care)—and, therefore, their programme acceptance—and decreasing the programme’s feasibility (e.g. formalisms, organisational workload, and low resources). Supplementary Material 1 summarises the observed relationships.

### Implementation process

The isPO programme includes many components and may be characterised as a complex intervention. All isPO components were implemented simultaneously at four different implementation sites, which may be challenging due to different contextual factors and different needs at the implementation sites [15]. Therefore, choosing adequate implementation strategies may be crucial [15]. Especially during the interviews and focus groups, many service providers referenced the implementation strategies and identified aspects that may have negatively affected the programme’s feasibility. However, they appreciated isPO quality management actions to optimise the programme according to the CNs’ needs. Two reappearing themes regarding implementation strategies were (1) the initial top-down programme development and implementation with little participation possibilities for the service provider and (2) the perceived lack of analysis of pre-existing care structures and contextual factors at the implementation sites. In the service providers’ opinion, this may have led to diverse challenges during the programme’s implementation, as also described by other researchers regarding the implementation of complex interventions [39, 49]. Including opportunities for early participation might have facilitated local tailoring, as proposed by others regarding complex interventions [39, 50]. Pre-design and pre-implementation stakeholder analyses are considered helpful in identifying factors hindering programme implementation [51, 52]. For example, in isPO, there appeared a bottleneck phenomenon regarding enrolment. Medical staff were not sufficiently involved in the implementation strategy, and there was little knowledge about the quality of interdisciplinary cooperation at the respective CNs. Further organisational factors, such as staff shortages and rotation systems, added to this. Early involvement of medical staff and actions to strengthen interdisciplinary cooperation between the oncological wards and PO service providers might have facilitated programme implementation. Our qualitative results suggest that service providers’ meaningfulness regarding the programme acted strongly, facilitating engagement and promoting programme implementation. At the same time, if individuals did not experience or show such meaningfulness, it negatively affected interdisciplinary cooperation, leading to more work for those with higher meaningfulness. Therefore, involving all stakeholders in the implementation process and applying strategies to increase individual meaningfulness may prevent implementation barriers later.

### The isPO programme during the COVID-19 pandemic

Like the overall German healthcare system, the isPO programme was affected by the COVID-19 pandemic. However, the qualitative results suggest that the new structures were flexible enough to adapt quickly to the new conditions. Working from home led to the restructuring of work processes for the service providers and allowed for more flexibility in patient care. However, some service providers also experienced impaired work-life balance and isolation. These disadvantages were also identified by other pandemic-related research [53, 54]. Those CNs that had more resources (e.g. rooms for isPO onco-guides) were faster in reinstating old processes (pre-pandemic), indicating how crucial contextual factors are for a system’s adaptability [55]. The qualitative results further suggest that facilitating factors appeared to be clarity and comprehensibility of one’s role in the programme, work routines, and good cooperation and social capital. Moreover, it appears important that information flow and team meetings (e.g. via digital quality circles) were maintained. Maintaining good communication and quality management was crucial for patient safety, as also described by other researchers [56, 57]. Overall, the isPO programme, including the isPO service providers, showed resilience towards the pandemic-related changes.

The perceived higher demand underlined the relevance of PO care. Further, patients who usually had care access barriers (e.g. due to their residence) could now be cared for via telephone or video. Studies have shown that some patients feel less stigmatised and more comfortable when receiving telephone/video-based therapy [58, 59]. Spatial distance may facilitate patients’ participation and engagement, especially when they experience anxiety, shame, or guilt [58, 59]. However, adapting to individual patients’ needs is still important since some prefer face-to-face sessions.

### Methodological strengths and limitations

The MMD enabled us to explain and supplement the quantitative results and gather in-depth data on the isPO programme’s care quality by collecting qualitative data.

Due to the small number of involved isPO service providers and not all participating in the surveys, our sample size was rather small. Therefore, we could mainly only analyse the data descriptively, which limits the generalisability. Further, there is a risk of bias when surveying service providers who are very engaged within the programme and therefore tend to assess it more positively [43]. However, we were able to gather differentiated data on the implementation context regarding work-related, organisational, and individual factors, allowing us to uncover possible associations between QoC and contextual factors, which might be helpful for future implementation actions. Moreover, the risk for biases, such as social desirability and common method or recall bias, must be considered when conducting surveys. Adding the survey of oncological staff was beneficial for gaining further insight into their assessments. The shorter questionnaire version appeared to enable us to gain a higher sample of these professional groups.

Qualitative data allowed us to obtain differentiated and in-depth information on the service providers’ experiences and attitudes regarding the isPO programme. By including all isPO-roles in the sampling and conducting across-network focus groups, we could capture diverse perspectives on the isPO programme regarding direct patient interaction, team structures and teamwork, interdisciplinary cooperation, facilitators and barriers, implementation strategies, and social and health system-related themes. The rich information we extracted from the qualitative data shows the potential of including service providers’ perspectives early and continuously in programme development, implementation, and optimisation, as proposed by others who recommend involving important stakeholders [15, 50, 52]. We conducted the interviews and focus groups via telephone and a videoconference tool. While it cannot be discounted that this affected the dynamic during data collection, we still received nuanced and comprehensive data.

## Conclusions

Overall, isPO service providers assessed the programme’s QoC positively. Further, they identified a few implementation barriers and optimisation needs, providing indications of the programme’s feasibility and possible fit for routine care. Including participative elements in the implementation or even development phase may support intervention designers in adapting it to different implementation sites’ needs. While the isPO programme is highly complex with its various interacting programme components, service providers’ experience with the stepped and needs-centred care approach indicates that its underlying concept is recommendable for routine care. However, persistent programme optimisations should be enabled and conducted within structured quality management.

## Supplementary Information

Below is the link to the electronic supplementary material.

Supplementary Material 1

## Data Availability

The datasets generated and analysed during this study are not publicly available due to participants’ consent restricting data use to the research team. However, they are available from the corresponding author upon reasonable request. Due to German data protection law, only de-identified data may be shared.
