# Supplementary material for: Service providers’ perspectives on the quality of care of a new complex psycho-oncological care programme in Germany – mixed methods external evaluation results
Source: BMC Health Serv Res. 2026 May 30;26:773. doi: 10.1186/s12913-026-14854-y (PMC13222498; doi:10.1186/s12913-026-14854-y)

**Supplementary Material**

**Additional File 1**


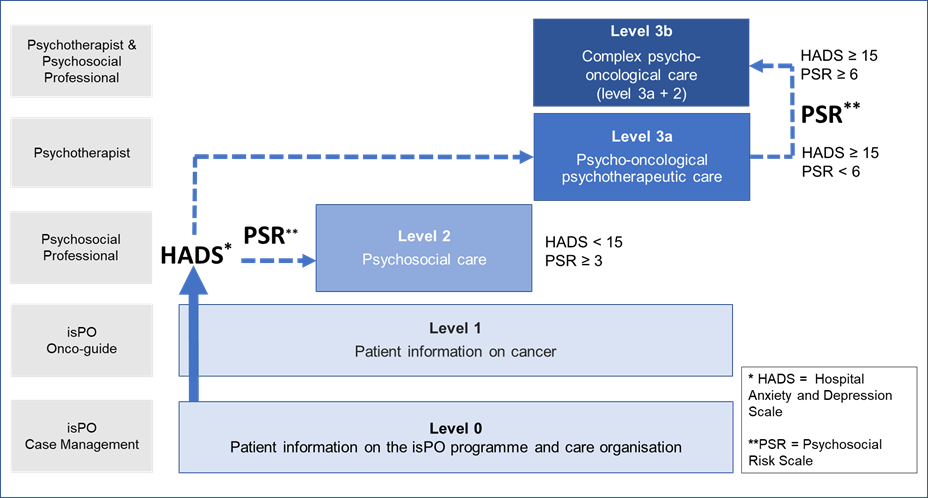


*Figure A. The isPO programme’s stepped care concept. Adapted from Kusch et al. and Salm and Cecon et al.*

Kusch M, Labouvie H, Schiewer V, Talalaev N, Cwik JC, Bussmann S, et al. Integrated, cross-sectoral psycho-oncology (isPO): a new form of care for newly diagnosed cancer patients in Germany. BMC Health Serv Res 2022. doi:10.1186/s12913-022-07782-0.

Salm S, Cecon N, Jenniches I, Pfaff H, Scholten N, Dresen A, Krieger T. Conducting a prospective evaluation of the development of a complex psycho-oncological care programme (isPO) in Germany. BMC Health Serv Res. 2022;22:531. doi:10.1186/s12913-022-07951-1.

**Additional File 2**

*Table A. Overview and specifics on the scales and items used to assess contextual variables from the isPO service provider’s perspective.*

| **Variable** | **Scale and item specifics** | **Answering scale** | **Example item** |
| --- | --- | --- | --- |
| Sufficient resources for the isPO | Three items, self-developed, on human, time, and financial resources. | Four-point scale from ‘not at all’ to ‘totally’. | To what extent are the following resources in your care facility sufficient to implement the ispO concept?  - Financial resources |
| Information needs* | Three items, self-developed, on work tasks, care processes, and project goals. | Four-point scale from ‘does not apply’ to ‘applies’. | When working in the isPO programme, I would have liked more information about my work tasks / my role in the programme |
| Planning deficits [31] | Reduced to two items on planning deficiencies and impairments due to unclear responsibilities and requirements | Four-point scale from ‘does not apply’ to ‘applies’. | In my work in the isPO programme, I have to cope with planning shortcomings in other areas. |
| Organisational workload | One self-developed item. | Four-point scale from ‘do not agree at all’ to ‘totally agree’. | The organisational effort within the programme is high. |
| Restrictions through isPO* | One self-developed item. | Four-point scale from ‘do not agree at all’ to ‘totally agree’. | The work that falls to me within the programme limits me in my everyday work tasks. |
| Changes in work conditions through isPO | One self-developed item. | Five-point scale from ‘very much worsened’ to ‘very much improved’. | To what extent have your working conditions changed as a result of the project? |
| Work-related sense of coherence [32] | Nine items on work-related comprehensibility, manageability, and meaningfulness. | Seven-point scale with changing poles (e.g. from ‘structured’ to ‘chaotic’ or ‘predictable’ to ‘unpredictable’). | How do you personally feel about your current work or work situation in general? |
| Effort Reward Imbalance Scale [33] | Sixteen items with three subscales on overcommitment, effort, and reward. | Four-point scale from ‘do not agree at all’ to ‘totally agree’. | It often happens that I think about work problems when I wake up.  Due to the high workload, there is often great time pressure.  When I think of all the achievements and efforts made, I think the recognition I received is appropriate. |
| Cooperation [31] | Four items on within and between professions. | Four-point scale from ‘do not agree at all’ to ‘totally agree’ and one non-response category (‘I cannot judge’). | Cooperation within the narrower working area is good. |
| Trustful organisation [31] | Four items. | Four-point scale from ‘do not agree at all’ to ‘totally agree’. | In our institution, we have trust in each other. |
| Social backing [34] | Three items. | Five-point scale from ‘does not apply at all’ to ‘totally applies’. | I can rely on my colleagues when things get difficult at work. |
| Open communication [31] | Four items. | Four-point scale from ‘do not agree at all’ to ‘totally agree’. | In our institution, problems are addressed openly. |
| Organisational readiness to change [35] | Twelve items on change commitment and change efficacy.  Translated into German | Five-point scale from ‘do not agree at all’ to ‘totally agree’. | Persons who work here…  … are determined to implement this change.  … can get people invested in implementing this change. |
| Innovation climate [31] | Seven items. | Four-point scale from ‘do not agree at all’ to ‘totally agree’. | In our institution, we are motivated to bring in new ideas. |
| Resistance to change [36] | Nine items on routine seeking and short-term focus. | Six-point scale from ‘do not agree at all’ to ‘strongly agree’. | I generally consider change to be negative.  I often feel a bit uncomfortable about change, even if it may improve my life. |
| Personality traits [37] | Ten items on extraversion, agreeableness, conscientiousness, neuroticism, and openness. | Five-point scale from ‘does not apply at all’ to ‘totally applies’. | I am outgoing and sociable.  I am generally trusting and believe in the good in people.  I do a thorough job.  I get nervous easily.  I have an active imagination. |
| *****these items which represent contextual factors, were measured in both, the main survey and the additional survey of medical staff | | | |

**Additional File 3**

*Table B. Overarching guiding questions of the interview guidelines.*

| **Guiding questions for the telephone interviews with the head psycho-oncologists** |
| --- |
| What is your task and role in isPO?  How do you evaluate the role of the clinical head of isPO? |
| How do you experience communication with the project staff involved in developing the care concept and the care processes? |
| How do you experience the exchange at the quality workshops? |
| How would you explain isPO to a colleague? |
| What distinguishes isPO from other psycho-oncological care programmes? |
| How do you assess the maturity of the care concept for the respective care levels? |
| Care level 3* in the treatment manual is important for your work.  How would you rate the isPO treatment manual specifically developed for care level 3? |
| How do you experience the utilisation of CAPSYS^2020^ in your daily work? |
| How do you assess the changes that have taken place through isPO? |
| Considering the isPO programme as a whole, how would you end the following sentence?  I find isPO ... |
| In your opinion, what would still have to be changed in the care concept or the care processes in order to be able to implement the programme nationwide in Germany? |
| What would you wish for isPO from your profession’s point of view? |
| Is there anything else striking / problematic / good / important that you would like to tell us about the isPO project that has not come up? |
| **Guiding questions for the two focus groups*** |
| What do you associate with isPO? |
| How would you rate isPO in the course of the project so far?  *Note: participants were asked to answer regarding different project phases (normalisation phase, Corona and status quo)* |
| How do you experience the implementability of isPO in your care network in regard to facilitating factors?  *Note: participants were asked to answer regarding the normalisation phase)* |
| How do you experience the implementability of isPO in your care network in regard to hindering factors?  *Note: participants were asked to answer regarding the normalisation phase)* |
| How do you experience the implementability of isPO in your care network throughout Corona?  *Note: participants were asked to answer regarding facilitating and hindering factors.* |
| What would it take for isPO to be transferred to routine care?  *Note: participants were asked to answer regarding different perspectives (patients, isPO service providers, biomedical oncological service providers, administration, isPO-designers, society)* |
| *The questions were also used for the virtual interview of one person that could not attend one of the focus groups. |

**Additional File 4**

*Figure B. Head codes of the coding system for interviews with isPO head psycho-oncologists.*


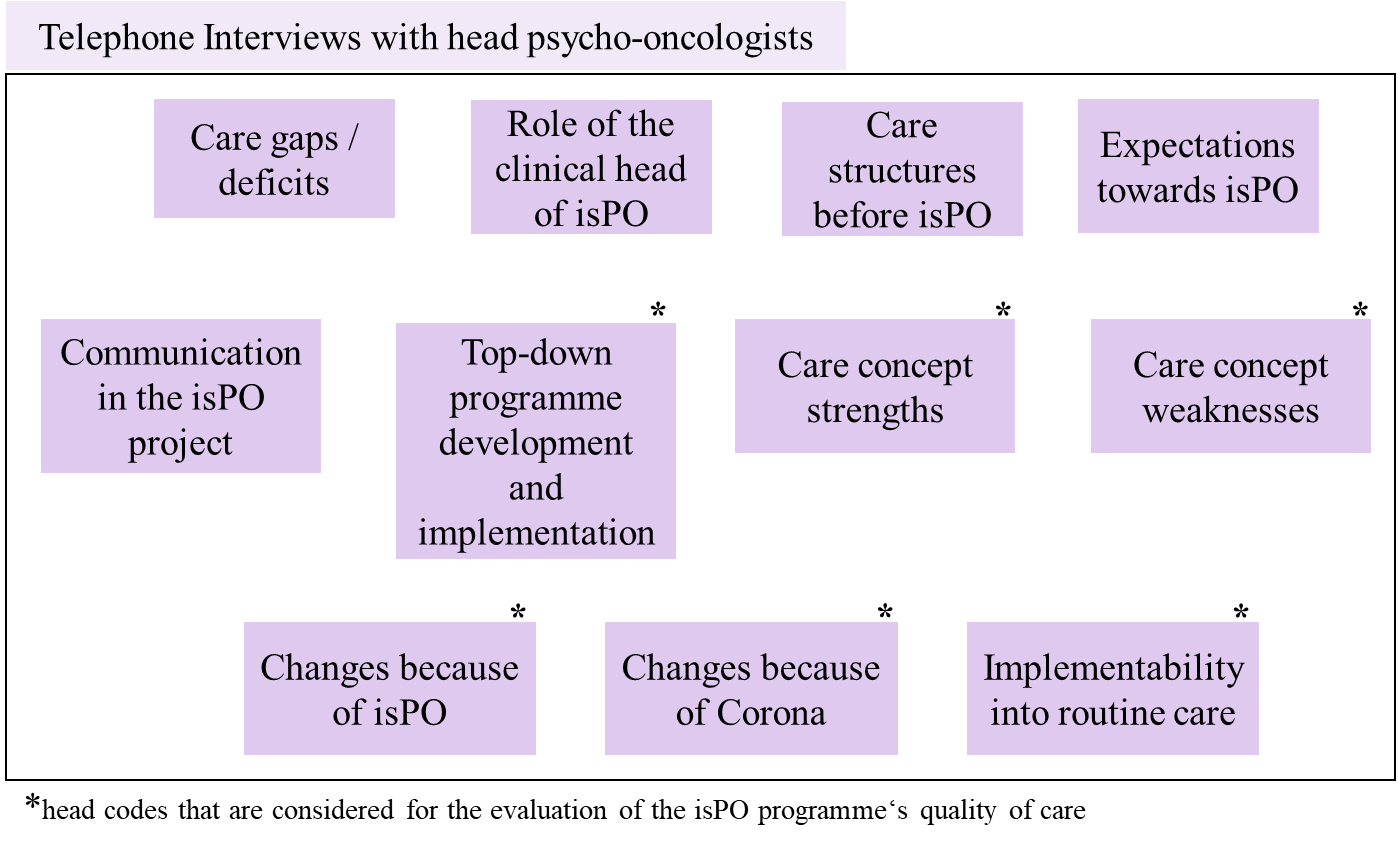


*Table C. Coding system (of relevant codes) for the telephone interviews with the head psycho-oncologists.*


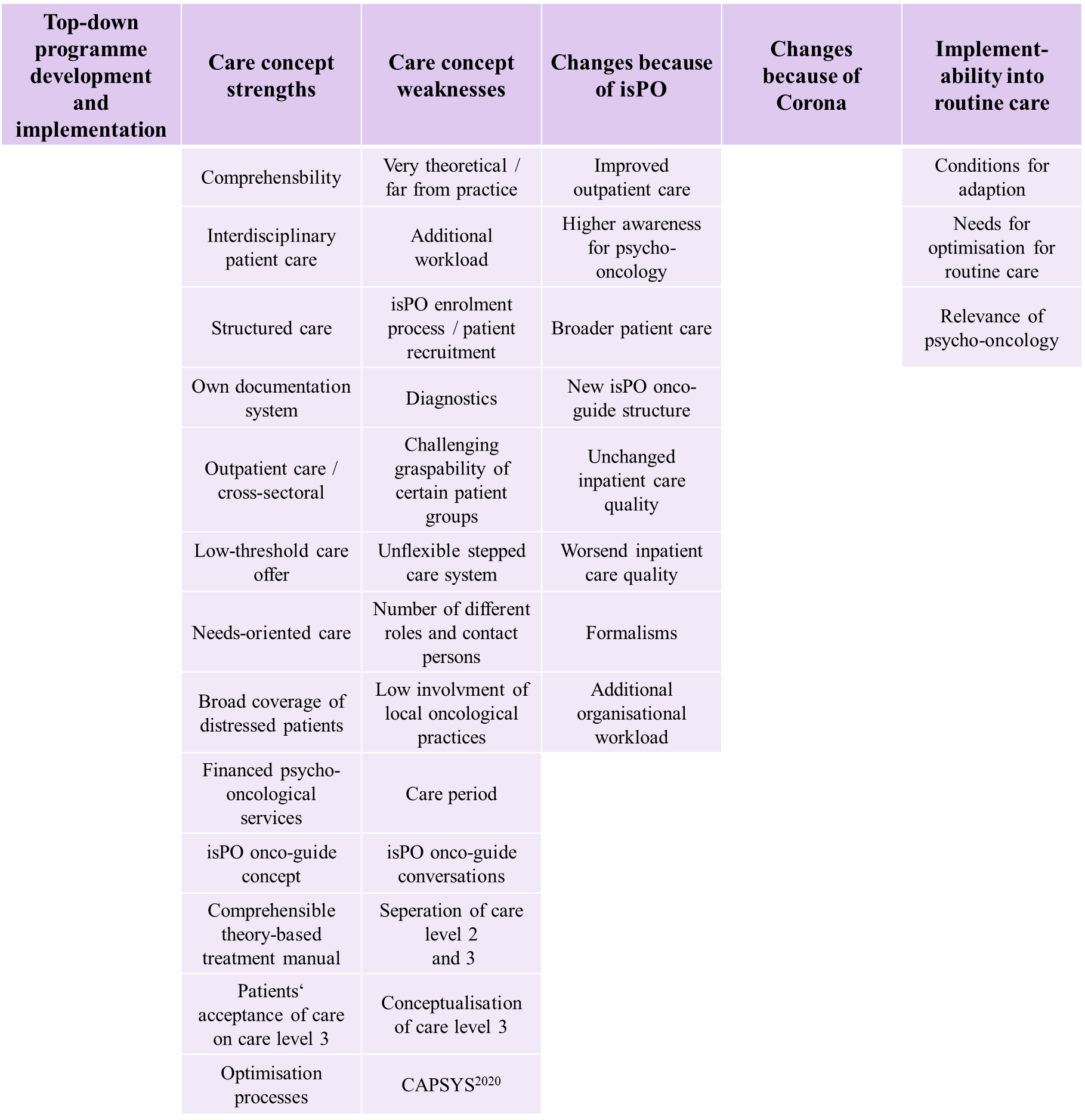


**Additional File 5**

*Table D. Coding system for Focus Group 1.*


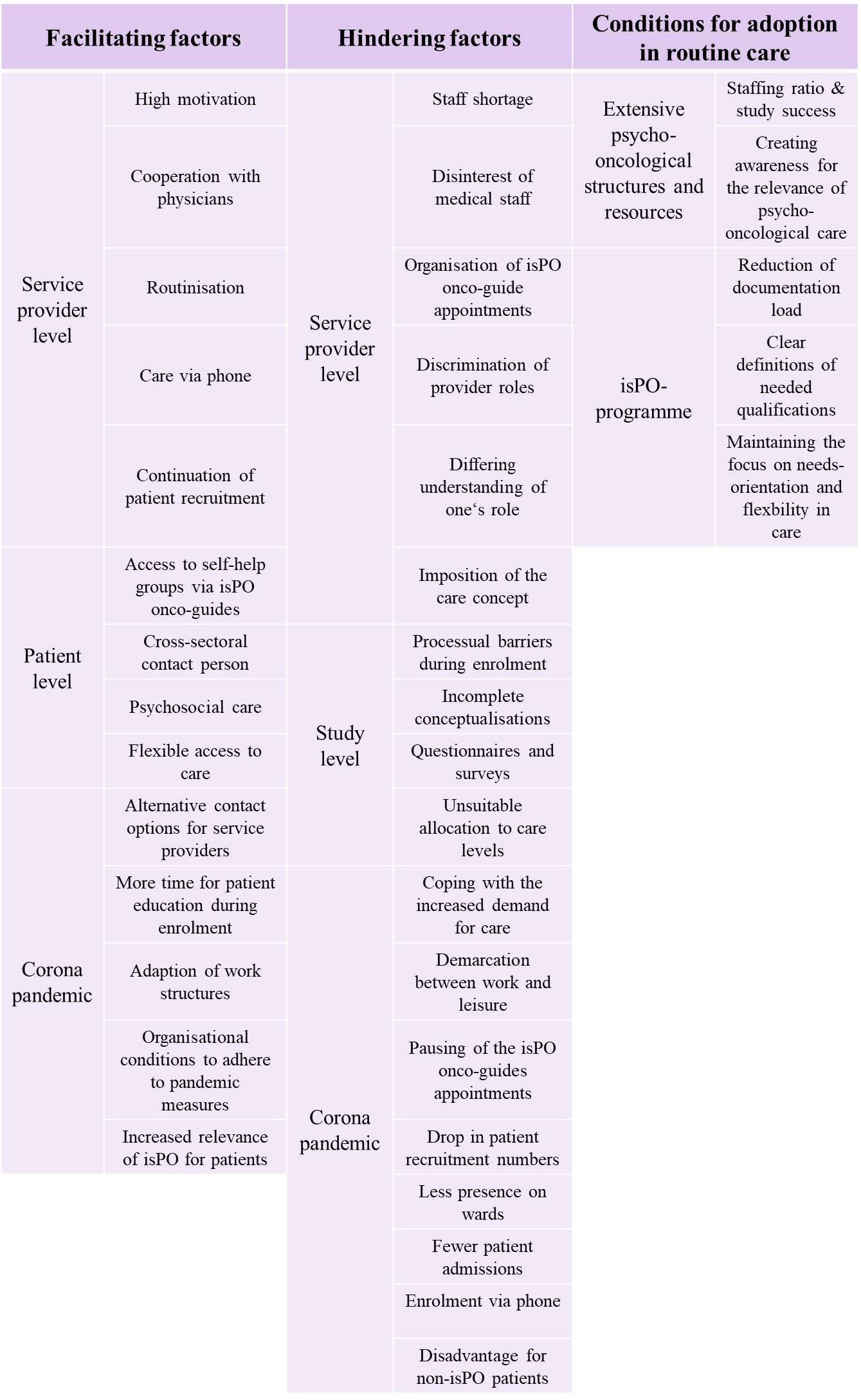


Table E. *Coding system for Focus Group 2.*


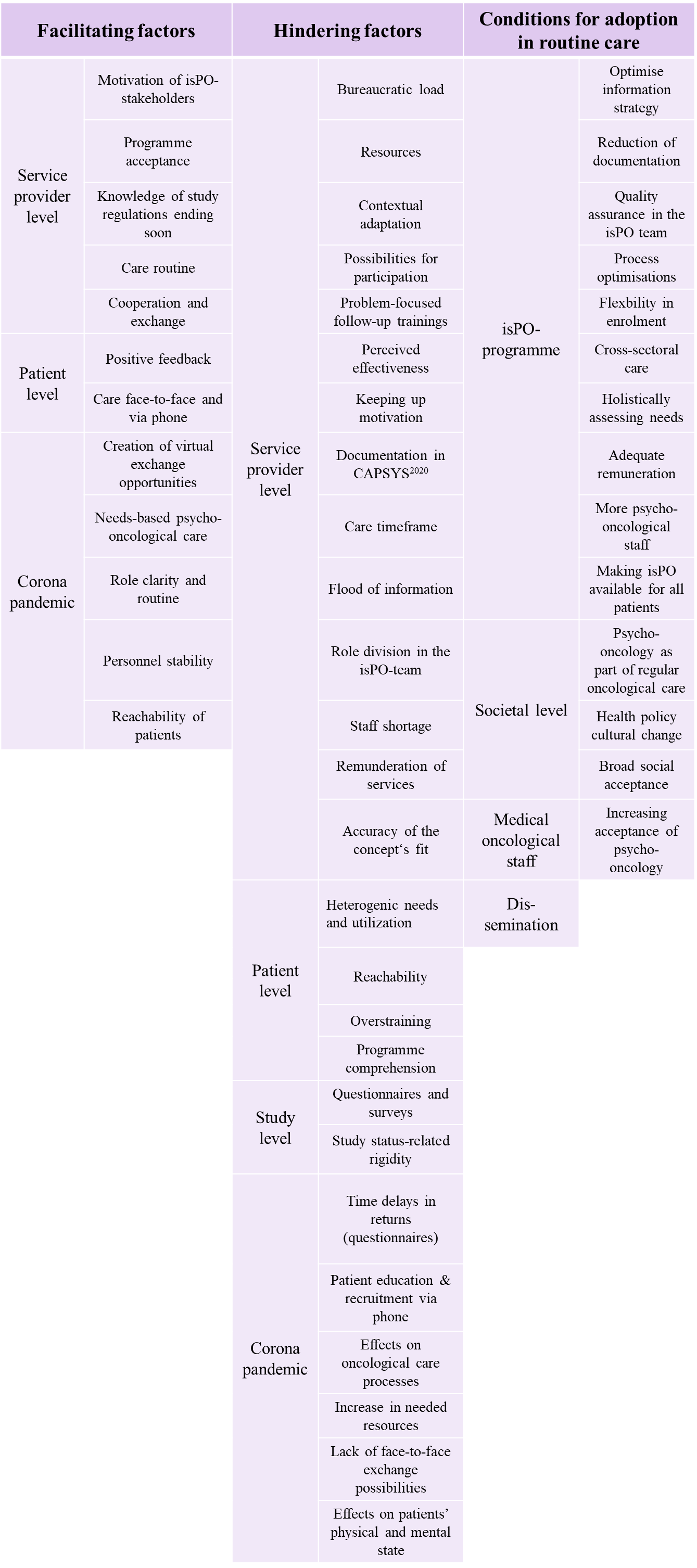


*Continuation of Table D – Subcodes of hindering factors.*


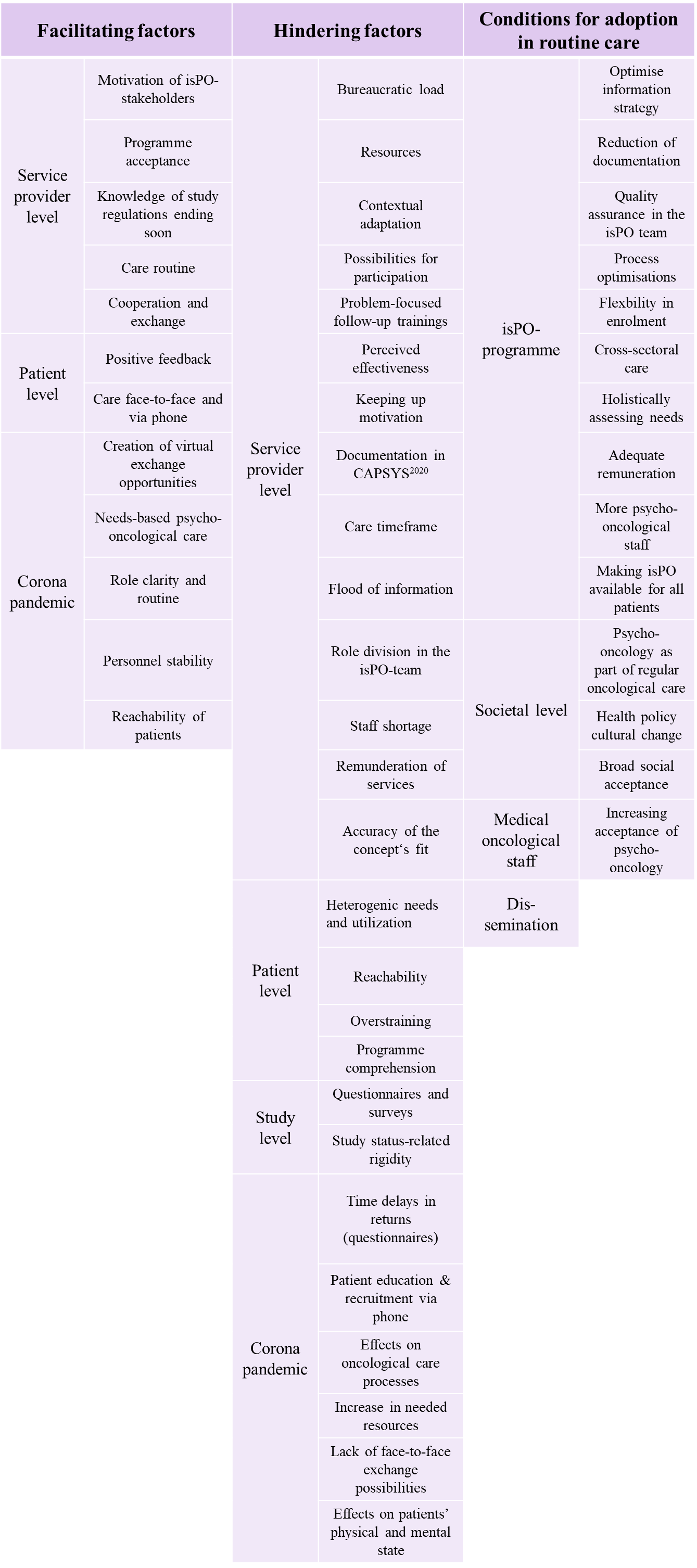


**Additional File 6**

*Figure C. Observed relationships between the complex interventions programme isPO, implementation strategies, feasibility, programme acceptance, contextual and individual factors, quality management, perceived challenges, and patient benefits.*


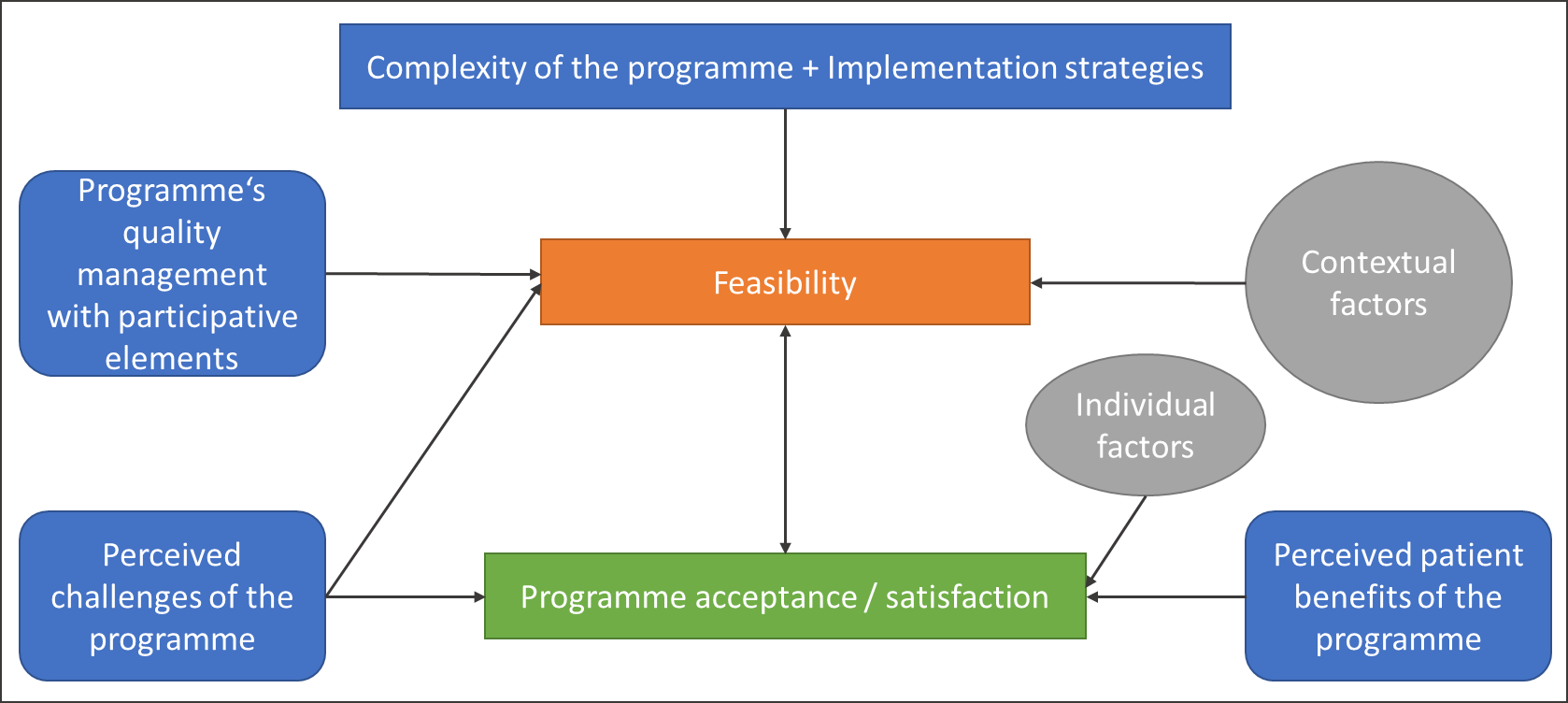

Supplement: Supplementary file 1 — Supplementary Material 1 [file 12913_2026_14854_MOESM1_ESM.docx]
